# Patient‐specific independent 3D GammaPlan quality assurance for Gamma Knife Perfexion radiosurgery

**DOI:** 10.1120/jacmp.v14i1.3949

**Published:** 2013-01-07

**Authors:** Maria Mamalui‐Hunter, Sridhar Yaddanapudi, Tianyu Zhao, Sasa Mutic, Daniel A. Low, Robert E. Drzymala

**Affiliations:** ^1^ University of Florida Proton Therapy Institute Jacksonville FL; ^2^ Department of Radiation Oncology Washington University in St. Louis School of Medicine St. Louis MO; ^3^ Department of Radiation Oncology University of California Los Angeles CA

**Keywords:** QA, Gamma Knife Perfexion, dose calculation, GammaPlan, ray tracing

## Abstract

One of the most important aspects of quality assurance (QA) in radiation therapy is redundancy of patient treatment dose calculation. This work is focused on the patient‐specific time and 3D dose treatment plan verification for stereotactic radiosurgery using Leksell Gamma Knife Perfexion (LGK PFX). The virtual model of LGK PFX was developed in MATLAB, based on the physical dimensions provided by the manufacturer. The ring‐specific linear attenuation coefficients (LAC) and output factors (OFs) reported by the manufacturer were replaced by the measurement‐based collimator size‐specific OFs and a single $\text{LAC}=0.0065\;{\text{mm}}^{-1}$. Calculation depths for each LGK PFX shot were obtained by ray‐tracing technique, and the dose calculation formalism was similar to the one used by GammaPlan treatment planning software versions 8 and 9. The architecture of the QA process was based on the in‐house online database search of the LGK PFX database search for plan‐specific information. A series of QA phantom plans was examined to verify geometric and dosimetric accuracy of the software. The accuracy of the QA process was further evaluated through evaluation of a series of patient plans. The shot time/focus point dose verification for each shot took less than 1 sec/shot with full 3D isodose verification taking about 30 sec/shot on a desktop PC. GammaPlan database access time took less than 0.05 sec. The geometric accuracy (location of the point of maximum dose) of the phantom and patient plan was dependent on the resolution of the original dose matrix and was of the order of 1 dose element. Dosimetric accuracy of the independently calculated phantom and patient point (focus) doses was within 3.5% from the GammaPlan, with the mean=2.3% and SD=1.1%. The process for independent pretreatment patient‐specific Gamma Knife Perfexion time and dose verification was created and validated.

PACS numbers: 87.53.Bn; 87.55.‐x; 87.56.Bg

## I. INTRODUCTION

Leksell Gamma Knife (Elekta Instruments AB, Stockholm, Sweden) stereotactic radiosurgery (SRS) treatments are widely prescribed for a variety of benign and malignant brain abnormalities. Leksell Gamma Knife (LGK) is a major treatment modality for single and multiple brain metastases, arteriovenous malformations, and trigeminal neuralgia. Given the fact that most of the LGK treatments are single‐dose SRS, independent dose calculations are needed to reduce likelihood of serious errors. Commercial solution for independent dose calculation was not available for the Gamma Knife Perfexion (LGK PFX) introduced in 2007 and its GammaPlan treatment planning software versions 8 and 9 that were installed at our institution in 2008. The importance of independent verification of GammaKnife treatments has been described in recent publications,^(^ ^1^ ^,^ ^2^ ^)^ which concentrated the quality assurance process of the source configuration and point dose verification. This work presents the irradiation time and 3D dose independent verification technique. The process can be fully incorporated into the treatment workflow to ensure accuracy of patient treatments.

The LGK PFX has a series of significant hardware differences compared to its LGK predecessors. Relevant to this discussion is that the LGK PFX has 192 Co‐60 sources, arranged in eight sectors (polar angle incremented) and five rings (increment along the patient superior‐inferior (SI) axis). An LGK PFX sector can be either in open or blocked state. The 24 sources in each sector are designed to independently slide in the SI direction along the ‘cone‐shaped’ shielded surface of the tungsten collimator to align with apertures that project 16, 4, and 8 mm diameter beams at the unit center point or ‘beam focus’. The more detailed information on the geometry of the new version of the Gamma Knife can be found in multiple publications.^(^ ^1^ ^–^ ^3^ ^)^ As in the previous versions of Gamma Knife, highly conformal dose distributions can be generated by a combination of beam apertures, treatment times, head angle, and patient shifts relative to the system isocenter. Patient irradiation at each individual treatment position is called a “shot”.

## II. MATERIALS AND METHODS

The virtual model of LGK PFX was built using MATLAB (MathWorks Inc., Natick, MA) programming environment, based on the physical dimensions of the machine supplied by the manufacturer. After a series of phantom studies, a measurement‐based collimator size‐specific OFs^(^ ^4^ ^)^ were employed. Output factors were 1, 0.92, and 0.81 for 16, 8, and 4 mm collimator sizes, respectively; and a single LAC=0.0065 /mm.

**Figure 1:**
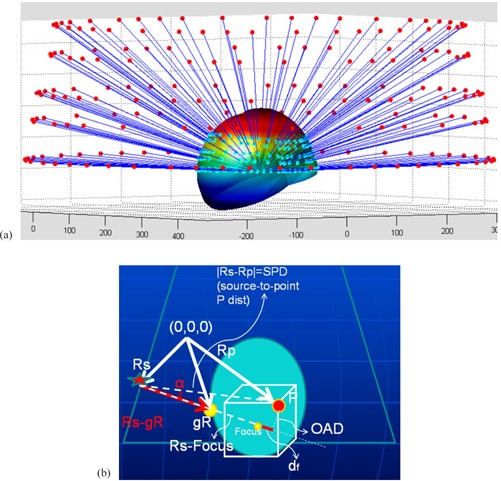
Ray‐tracing in case of gamma angle 70° (a) (cyan dots: points of beam surface entry); dose calculation geometry (b): Rs, gR, and Rp are radius vectors of an arbitrary Co‐60 source, the beam entry point along the skull surface and the arbitrary calculation point P, respectively. OAD is the effective off‐axis distance used for the off‐axis beam profile scaling. The angle α between the beam axis and the source‐to‐point P line is shown.

The patient skull surface was reconstructed based on the standard set of Leksell skull scaler measurements. In order to obtain the tissue depths for each LGK PFX beam, a simple ray‐tracing routine that picks a parallel ray to each source‐to‐focus beam axis (Fig. 1(a)) was used.

The dose calculation formalism was similar to the one used by GammaPlan treatment planning software^(^ ^3^ ^)^ (Fig. 1(b)) for the geometry corresponding to the Eq. (1):

(1) $${\left(\frac{dD}{dt}\right)}_{i}(P)={\left(\frac{dD}{dt}\right)}_{calibration,16}\times \frac{1}{192}\times OF_{c}\times \frac{e^{-\mu (d-d_{f}-R_{calibration})}}{{\left(1-\frac{d_{f}}{vSFD_{i}}\right)}^{2}}\times OAR\left(\frac{OAD_{f}}{\left(1-\frac{d_{f}}{SSD_{i}}\right)}\right)$$

Here $\left({\frac{dD}{dt}}_{i}\right)(P)$ is the dose rate at the location *P* resulting from the ith source, ${\left(\frac{dD}{dt}\right)}_{calibration,16}$ is the current calibration dose rate, which is further normalized to the number of machine sources (192), and the subscript index indicates the 16 mm collimator size used in calibration setup. The output factor $OF_{c}$ of the ith source is collimator size‐specific, as described earlier. The attenuation is accounted for as the factor $e^{-\mu (d-d_{f}-R_{calibration})}$, where *d* is the depth of the focus point measured along the ith beam axis, $d_{f}$ is the signed difference between the focus depth and the depth of the point *P* projected onto the ith beam axis, and $R_{calibration}$ is the depth of the focus point in the calibration geometry (80 mm). The inverse square effect is taken into account by the factor ${\left(1-\frac{d_{f}}{vSFD_{i}}\right)}^{-2}$, where $vSFD_{i}$ is the collimator size and ring‐specific virtual source‐to‐focus distance adopted from the manufacturer‐provided information.^(^ ^3^ ^)^ The off‐axis beam profile (off‐axis ratio, or OAR), pertinent to the ith source, was scaled according to the beam divergence as follows: $OAR\left(OAD_{f}/\left(1-d_{f}/SSD_{i}\right)\right)$, where $OAD_{f}$ is the off‐axis distance at the depth *d* of the focus point, and $SSD_{f}$ is the source‐to‐surface distance corresponding to the ith source and calculated as the difference between $vSFD_{i}$ and the depth of the point *P* projected onto the ith beam axis.

**Figure 2:**
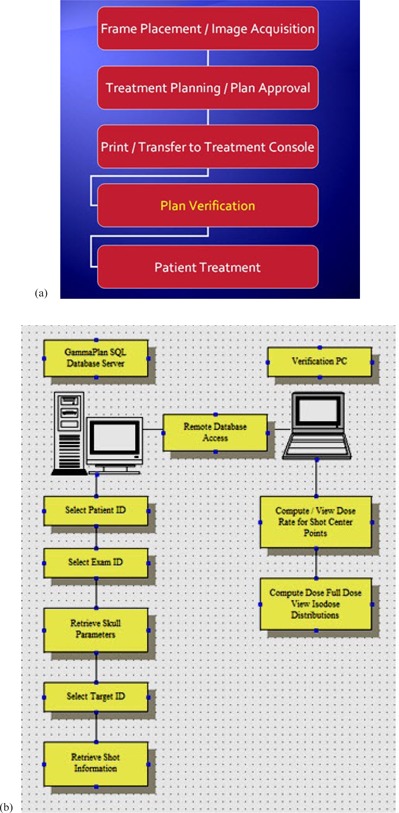
Workflow (a) of the Gamma Knife patient information; steps (b) of the plan verification process.

The general scheme of the clinical workflow with the incorporated verification process is shown in Fig. 2(a). The graphical user interface of the GammaPlan verification tool (GPVT) was developed in‐house using MATLAB programming environment. Once the treatment plan is completed in GammaPlan, it is approved and sent to the machine for treatment. At this time, the operator launches the GPVT program to retrieve treatment‐related information such as location and number of the targets and shots, and patient skull coordinates from the PostgreSQL database that backbones the Leksell GammaPlan treatment planning system. A connection was established between the GPVT program and the database via a PostgreSQL Java Database Connectivity (JDBC) driver obtained from PostgreSQL's official website (www.postgresql.org). Five tables: ‘patients’, ‘examinations’, ‘skulls’, ‘targets’, and ‘shots’ were cross‐linked to fetch the delivered treatment parameters by specifying the patient's name and ID. This process is shown in Fig. 2(b). The operator selects the examination and current plan used for treatment in case multiple plans exist for the patient. The program performs the calculation based on the algorithm explained above and outputs the result in an HTML format. The GPVT program has been developed in such a way that the PDF treatment plan that is printed out by GammaPlan can be parsed, and the results from the GPVT program are compared against their counterpart from GammaPlan.

The calculations were performed using the following hardware: Dell Precision T5500, Intel Xeon CPU, E5520 @ 2.27 GHz 2.26 GHz, 12 GB RAM.

A series of QA phantom plans was examined to verify geometric and dosimetric accuracy of our software. This was followed by calculation of a series of patient plans, which aimed at establishing action criteria for our QA process. We focused on making sure that our GPVT software works through a comprehensive set of spherical phantom and patient clinical plans (see Table 1 for representative subset of the patient population and diagnoses treated at our facility). It was important to show consistent agreement with the GammaPlan focus dose output within the accepted clinical tolerance (±3%) and action (±5%) limits. The goal was to ensure that the extremes caused either by treatment planning software malfunction or operator error are filtered out.

**Table 1:** Representative summary of the clinical use of the GPVT.

*Patient #*	*Diagnosis*	*Number of Shots*	*Time (sec)*
Patient 1	Multiple Metastasis	11	20.99
Patient 1	Multiple Metastasis	1	2.04
Patient 1	Multiple Metastasis	5	9.59
Patient 2	Multiple Metastasis	4	7.93
Patient 2	Multiple Metastasis	3	5.83
Patient 3	Multiple Metastasis	2	4.02
Patient 4	Trigeminal Neuralgia	1	2.06
Patient 5	Other Benign Tumor	19	37.96
Patient 6	Multiple Metastasis	10	18.84
Patient 6	Multiple Metastasis	2	4.01
Patient 7	Acoustic Schwannoma	14	27.73
Patient 8	Single Metastasis	6	11.26
Patient 9	Acoustic Schwannoma	12	23.76
Patient 10	Single Metastasis	9	17.29

## III. RESULTS

As mentioned in the previous section, Table 1 shows the representative subset of the clinical use of the GPVT software in our Gamma Knife Center.

The shot time and focus point dose verification took less than 1 sec per shot, with the full 3D isodose verification taking about 30 sec per shot. GammaPlan database access time was less than 0.05 sec. The geometric accuracy (i.e., location of the point of maximum dose) of the phantom and patient plan was dependant on the resolution of the original dose matrix and was of the order of 1−1.5 times the dose matrix voxel (“dosel”). During the initial evaluation stage, the dosimetric accuracy of independently calculated phantom and patient point dose was within 3%−3.5%, compared to GammaPlan version 8.3.1, with the mean=2.3% and SD=1.1%.

**Figure 3:**
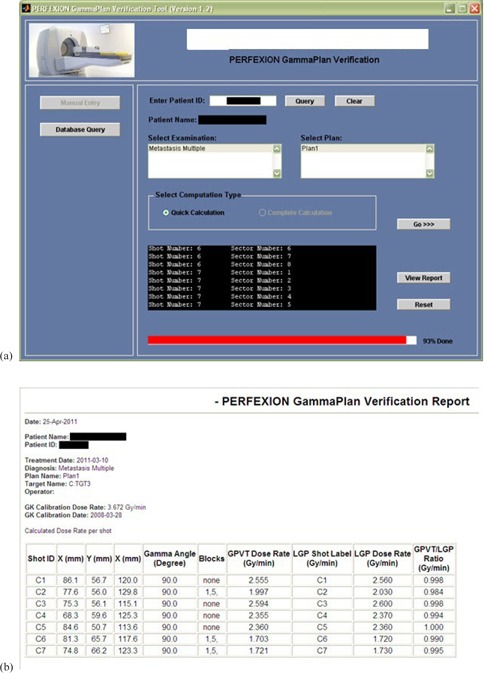
Graphical User Interface (a) of the GammaPlan verification tool (GPVT); HTML report (b) of the results obtained from GPVT.

The graphical user interface of the GPVT software and an example report of independent dose calculation for a sample clinical case are demonstrated in Figs. 3(a) and 3(b). The statistics of the clinical patient pretreatment focus dose verification is presented in Table 2 for 15 patient cases: the average calculated‐to‐planned dose ratio over the planned number of shots, standard deviation, and the range of calculated ratios of GPVT to GammaPlan focus dose. All of the GPVT calculated average doses to the target deviated from the GammaPlan dose within 3% range, with 6 shot doses out of total 203 shots examined being out of ±5% range.

**Table 2:** Statistics of the clinical plan dose verification: average over the number of shots, standard deviation, and the range of calculated ratios of GPVT to GammaPlan focus dose.

			*GPVT Calculated vs. GammaPlan*		
*Patients*	*Number of Shots*	*Average*	*S.D.*	*Max*	*Min*
1	15	0.998	0.008	1.006	0.983
2	7	1.015	0.005	1.019	1.005
3	18	1.002	0.008	1.018	0.991
4	25	1.018	0.018	1.049	0.988
5	17	0.967	0.013	0.984	0.947
6	12	0.990	0.009	0.999	0.969
7	3	0.981	0.003	0.984	0.978
8	14	1.021	0.019	1.038	0.960
9	30	0.978	0.014	1.021	0.955
10	13	0.996	0.004	1.002	0.993
11	18	1.026	0.012	1.048	1.008
12	6	0.973	0.003	0.977	0.968
13	2	0.979	0.003	0.981	0.977
14	12	0.956	0.010	0.976	0.943
15	11	0.997	0.008	1.008	0.983

**Figure 4:**
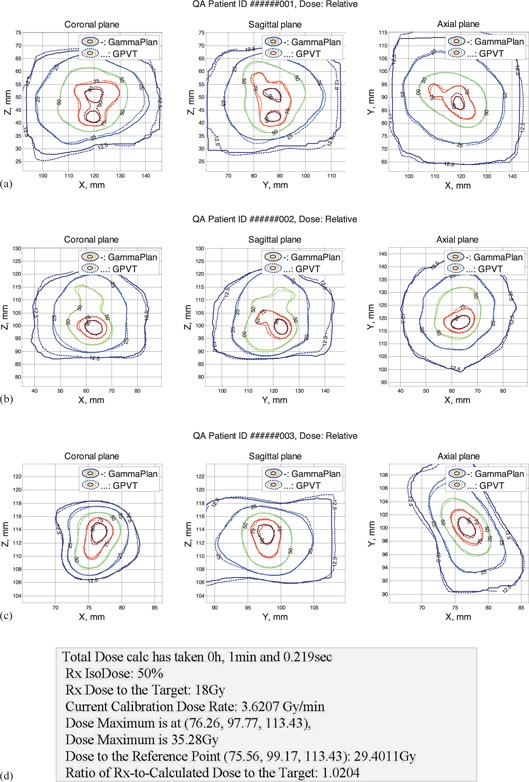
Full dose calculation results (patient information omitted): (a)–(c) isodose comparison display for three sample clinical plans; (d) sample dose comparison output.

Figures 4 (a) to 4(c) show isodose comparison in axial, sagittal, and coronal planes of the two dose matrices: one, calculated by GammaPlan, and the second matrix calculated by our LGK PFX QA software. The contour graphs demonstrate the close proximity and similarity of shape of 12.5%, 25%, 50%, 75%, and 90% isodose lines. Figure 4(d) shows the example of additional dose information printed out for each QA run, which also reveals a reasonably good agreement between the independently calculated and the GammaPlan dose.

## IV. DISCUSSION

Among likely sources of the 3D dose discrepancies between the GammaPlan and an independent dose verification QA software such as GPVT, several can be pointed out: the difference in skull surface interpolation between the vendor's and our MATLAB routine; and necessary interpolations performed in order to combine the multiple shot doses together in a single matrix. The former can be eliminated in situations where the vendor software is switched to using the patient CT numbers for rendering the skull surface, which could be easily implemented in our QA program. As the measurement of the relative output factors presents a continuous challenge,^(^ ^4^ ^–^ ^6^ ^)^ building a reasonably fast Monte Carlo‐based dose calculation engine seems to be the likely solution for the comprehensive independent time and dose verification for Gamma Knife treatment.^(^ ^7^ ^,^ ^8^ ^)^ Unlike the simple ray‐tracing–based technique presented in this work, the Monte Carlo approach would be able to take into account the leakage and scatter contribution into the total delivered dose, which can have a noticeable effect in case of the smallest 4 mm collimator size, due to the lowest output.^(^ ^1^ ^,^ ^7^ ^)^ However, as a fast pretreatment verification of the patient treatment parameters, our QA routine ensures that focus dose is within the accepted clinical tolerance and/or action (±5%) limits, and the extreme cases of treatment planning software malfunction or operator error are red flagged.

Comparing the presented dose verification method to that used by Wright et al.^(^ ^2^ ^)^ shows that incorporating the interpolated skull surface model that is based on the skull scaler helmet measurements delivers similar results to the point dose calculations.

## V. CONCLUSIONS

An automated method of the patient‐specific treatment plan quality assurance for Leksell Gamma Knife Perfexion stereotactic radiosurgery is presented. The technique employs fast database access for the remote read‐only extraction of treatment specifications from the GammaPlan PFX patient SQL database over the local network. It enables independent treatment plan verification on a separate PC workstation so that the FDA approval of GammaPlan software is not compromised. The software independently computes and compares with the GammaPlan dose rates at selected points of interest, and computes and displays isodose distribution and the time for each shot for comparison with the GammaPlan results.

The LGK PFX QA code was validated using phantom and anonymous patient plans with clinically relevant agreement with the GammaPlan TPS version 8.3.1. The code workflow is tested to be fast and suitable for the pretreatment plan verification.
